# The prevalence of vertebral fractures in spondyloarthritis: relation to disease characteristics, bone mineral density, syndesmophytes and history of back pain and trauma

**DOI:** 10.1186/s13075-015-0809-9

**Published:** 2015-10-22

**Authors:** Piet Geusens, Liesbeth De Winter, Dana Quaden, Johan Vanhoof, Debby Vosse, Joop van den Bergh, Veerle Somers

**Affiliations:** Hasselt University, Biomedical Research Institute, and Transnationale Universiteit Limburg, Diepenbeek, Belgium, Martelarenlaan 42, 3500 Hasselt, Belgium; Division of Rheumatology, Department of Internal Medicine, CAPHRI School for Public Health and Primary Care, Maastricht University Medical Center, Maastricht, P.O. Box 616, 6200 MD Maastricht, The Netherlands; ReumaClinic, Genk, Bretheistraat 149, 3600 Genk, Belgium; Department of Internal Medicine, VieCuri Medical Centre, P.O. Box 1926, 5900 BX Venlo, The Netherlands; Department of Internal Medicine, NUTRIM School for Nutrition, Toxicology and Metabolism, Maastricht University Medical Centre, P.O. Box 616, 6200 MD Maastricht, The Netherlands

**Keywords:** Spondyloarthropathy, Bone mineral density, Vertebral fracture, Vertebral fracture assessment

## Abstract

**Introduction:**

An increased risk of vertebral fracture (VF) is one of the extra-articular manifestations of spondyloarthropathy (SpA). The prevalence of moderate to severe VFs visualized by radiography (Rx) in patients with SpA in daily practice is unknown until imaging of the full spine is available, as most VFs do not present with clinical signs and symptoms of an acute fracture.

**Methods:**

We evaluated the prevalence of VFs (>25 % loss in height) on available Rx and dual-energy X-ray absorptiometry (DXA) images in 390 consecutive patients with SpA in daily practice. We assessed their association with disease characteristics, bone mineral density, the modified Stoke Ankylosing Spondylitis Spinal Score, and history of trauma.

**Results:**

Forty-six patients (11.8 %) had Rx VF (56.4 % men, 93.5 % in the thoracic spine), and 44.5 % had multiple VFs. Compared with patients without VF, patients with VF were older (52.2 vs. 47.3 years, *p* < 0.01; range 25–84 years), had lower femoral neck T-scores (−1.1 vs. −0.7; *p* < 0.05), and had a marginally higher modified Stoke Ankylosing Spondylitis Spinal Score (11.7 vs. 7.0; *p* = 0.06). Among patients with VFs, 15.2 % had a history of trauma with acute back pain (*p* < 0.001 vs. no VF). The reliability of DXA for diagnosing radiographic VFs was high (κ 0.90).

**Conclusions:**

Moderate to severe VFs are found in more than 10 % of patients with SpA before the age of 40 years in 5 % of women and 9 % in men. Most VFs are located in the thoracic region, are related to low femoral neck bone mineral density and to stiffening of the spine, and are only rarely related to trauma history. DXA is a useful alternative for diagnosing VFs.

## Introduction

An increased risk of vertebral fracture (VF) is one of the extra-articular manifestations of ankylosing spondylitis (AS) [1, 2] and other spondyloarthropathies (SpAs) [3, 4]. In population studies, it has been shown that the risk of radiographic and clinical VFs is increased in patients with AS [3, 5–9], even early in the disease [6, 10], and is also increased in those with inflammatory bowel disease (IBD) [3] and psoriatic arthritis (PsA) [4]. The prevalence of VFs has been studied in several surveys including 22–158 patients with AS in whom the prevalence of radiographic VFs ranged from 1.4 % to 58.0 %. Nevertheless, criteria for patient selection were variable in these studies (consecutive patients or patients selected on the basis of disease activity, occiput to wall distance (OWD), sex or age, and including clinical VFs or systematic evaluated morphometric VFs), as were the criteria for VF diagnosis (variable thresholds of vertebral height loss) [10–14].

Based on the increased rate of VF in AS, imaging of the spine is advocated in cases of significant change in the course of the disease [15, 16]. However, most VFs do not present with the clinical signs and symptoms of an acute fracture and are overlooked when back pain is interpreted as a disease flare of spondylitis. No data are available on the relationship between history of trauma and acute or chronic back pain in SpA.

Imaging of the spine is most often performed with conventional radiography (Rx), but Vertebral Fracture Assessment (VFA) using dual-energy X-ray absorptiometry (DXA) technology is of increasing interest because of its low radiation dose and high negative predictive value (NPV) [17], and it has also been studied in AS [13, 18, 19].

We evaluated the presence of VF using Rx and VFA in a non-academic rheumatology practice in a large cohort of patients with SpA in relation to disease characteristics, bone mineral density (BMD), syndesmophytes, and history of back pain and trauma.

## Methods

### Study population

A total of 390 consecutive ambulatory patients with SpA who were seen by six rheumatologists in an ambulatory non-academic rheumatology clinic were included between July 2013 and December 2013.

Diagnoses of AS, undifferentiated SpA, PsA, and IBD were made by the treating rheumatologists according to the criteria set forth in national and international guidelines [20]. This study was approved by the medical ethics committee of Ziekenhuisnetwerk Antwerpen (Belgium), and all patients provided informed consent before participation.

### Bone mineral density

BMD was measured in the lumbar spine (anteroposterior projection), femoral neck, and total hip using DXA with standard procedures as recommended by the manufacturer (Lunar Prodigy Primo BX-1 L enCORE device, version 12.30; GE Healthcare Life Sciences, Chalfont St. Giles, UK). VFA imaging was performed using software provided by the manufacturer for this device.

### Radiographic assessment and scoring

Lateral images of the thoracic and lumbar spine were semiquantitatively evaluated according to the Genant score for anterior, middle, and posterior height using available digital Rx and VFA images [21, 22]. VFA was performed using a semiquantitative (SQ) technique. Each Rx and VFA image was inspected visually by the treating rheumatologist to decide whether it contained a fracture in any of the visualized vertebrae and assigned a grade based on the Genant SQ scale, where grade 2 (moderate) is a reduction of 26–40 % and grade 3 (severe) a reduction of over 40 %. In case of doubt, vertebral heights were measured using the digital measuring device supplied by the provider of the digital Rx system. Only vertebral deformities >25 % were considered as a VF (grades 2 and 3), as this degree of deformity increases the specificity compared with a lesser degree of deformity [23].

On lateral spine radiographs, the cervical, lumbar and thoracic vertebrae were scored using the modified Stoke Ankylosing Spondylitis Spinal Score (mSASSS) as 0 (normal), 1 (erosion, sclerosis, or squaring), 2 (obvious syndesmophyte), and 3 (total bone bridge) [16]. The mSASSS according to Assessment of SpondyloArthritis international Society includes scoring of radiographs at the cervical and lumbar spine (range 0–72). In addition, the same scoring method was also used for the thoracic spine, and additional mSASSS was calculated for both the thoracic spine (range 0–72) and total spine (range 0–144).

Each of the six experienced rheumatologists performed scoring of mSASSS on radiographs and VFs on the radiographs and VFAs of their own patients. They were all experienced and trained in scoring VF, as well as in patients with SpA, at the yearly bone curriculum meetings of the Osteoporosis Working Group of the Royal Belgian Rheumatology Society. Therefore, no intra- or interreader reliability was tested.

### Disease characteristics

Disease characteristics of patients included the Bath Ankylosing Spondylitis Disease Activity Index (BASDAI), Bath Ankylosing Spondylitis Functional Index (BASFI), Ankylosing Spondylitis Disease Activity Score (ASDAS), OWD, presence of human leukocyte antigen B27 (HLA-B27), and serum erythrocyte sedimentation rate (ESR) and C-reactive protein (CRP). All patients were asked about a history of falls and trauma and subsequent acute or chronic back pain.

### Statistical analysis

We report descriptive statistics and comparisons between patients with and without a VF using analysis of variance (ANOVA) for continuous variables and cross-tabulation for dichotomous variables.

Univariable and multivariable logistic regression analyses were used to estimate the effects of factors that were significantly different between patients with and without a VF on the likelihood [expressed as odds ratio (OR) with 95 % confidence interval (CI)] that participants had a radiographic VF.

A reliability analysis using the κ statistic was performed to determine consistency between VFA and Rx in diagnosing VF. For the diagnostic value of VFA in identifying radiographic VF, we calculated sensitivity, specificity, positive predictive value (PPV), and NPV. Calculations were performed using IBM SPSS version 22 software (IBM, Armonk, NY, USA).

## Results

### Study population

The basic characteristics and BMD of the 390 patients with SpA are shown in Table 1. These included 175 (44.9 %) men and 215 (55.1 %) women, 286 (73.3 %) of whom had AS, 40 (10.3 %) of whom had PsA, 18 (4.6 %) of whom had IBD, and 46 (11.8 %) of whom had undifferentiated SpA. The patients’ mean age was 47.9 years [standard deviation (SD) 11.9, range 21–84 years]. The mean time since first symptoms was 14.6 years (SD 10.7, range 1–60 years), and the mean time since diagnosis was 10.8 years (SD 9.9, range: 0–60 years). The mean difference between symptoms and diagnosis was 3.9 years (SD 5.8, range 0–33 years). HLA-B27 was present in 203 (62.8 %) of the 323 patients in whom this was evaluated. In addition, 168 (43.1 %) of the patients had undergone treatment with tumor necrosis factor blockers at the time of the survey.

**Table 1:** Basic characteristics of included patients with spondyloarthritis (*n* = 390)

Characteristics	Mean^a^	Range^b^
Age, yr	47.9 ± 11.9	21–84
Symptom duration, yr	14.6 ± 10.7	1–60
Disease duration, yr	10.8 ± 9.9	0–60
ESR, mm/h	9.0 ± 10.4	0–86
CRP, mg/L	3.8 ± 6.3	0–60
BASDAI	4.3 ± 2.3	0–10
BASFI	5.0 ± 8.0	0–83
ASDAS-ESR	2.2 ± 1.0	0–5
ASDAS-CRP	2.2 ± 1.1	0–5
mSASSS		
Cervical spine	3.7 ± 9.2	0–42
Thoracic spine	10.6 ± 20.4	0–72
Lumbar spine	3.9 ± 8.0	0–30
Cervical + lumbar spine	7.6 ± 15.9	0–72
Total spine	18.1 ± 34.4	0–144
T-score		
Lumbar spine	−0.2 ± 1.7	−4.3 to ±10.4
Femoral neck	−0.7 ± 1.1	−3.8 to ±4.7
Total hip	−0.4 ± 1.1	−3.2 to ±4.9

*ESR* erythrocyte sedimentation rate, *CRP* C-reactive protein, *BASDAI* Bath Ankylosing Spondylitis Disease Activity Index, *BASFI* Bath Ankylosing Spondylitis Functional Index, *ASDAS* Ankylosing Spondylitis Disease Activity Score, *mSASSS* modified Stoke Ankylosing Spondylitis Spinal Score

^a^Characteristics of patients with SpA are presented as mean absolute number ± standard deviation

^b^Range of absolute numbers for a given characteristic

**Fig. 1:**
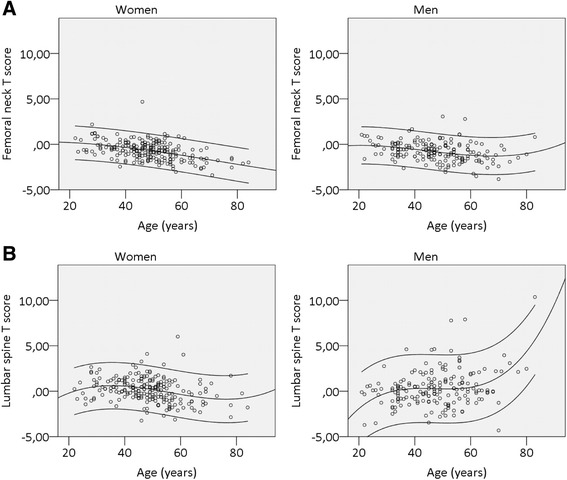
Correlation between bone mineral density (of the femoral neck and lumbar spine) and age. Bone mineral density of patients with SpA is represented as T-score. T-scores are shown in the (**a**) femoral neck and (**b**) lumbar spine for both women (*left*) and men (*right*) according to their age (years). *Lines* represent the 95 % confidence intervals

The majority of patients had normal BMD (T-score −1.0 or higher) in the spine (79.7 %) and in the femoral neck (59.5 %), whereas 17.2 % and 36.4 % had a T-score between −1 and −2.5, respectively. Only a minority of patients had a T-score −2.5 or lower (3.1 % in the spine and 4.1 % in the femoral neck). BMD decreased with age in the femoral neck in women (−0.46; *p* < 0.001) and men (−0.31; *p* < 0.001), as well as in the spine in women (−0.26; *p* < 0.001), but it increased with age in the spine in men (0.27; *p* < 0.001) (Fig. 1).

The classical mSASSS (cervical + lumbar spine) was 7.6 (SD 15.9, range 0–72). The mSASSS for the thoracic spine was 10.6 (range 0–72), resulting in a total spine mSASSS score of 18.1 (SD 34.4, range 0–144). mSASSS >0 were found in the cervical spine in 30.1 % of patients, in the thoracic spine in 43.6 %, in the lumbar spine in 38.2 %, in the cervical and lumbar spine combined in 45.1 %, and in the total spine in 55.1 %.

**Fig. 2:**
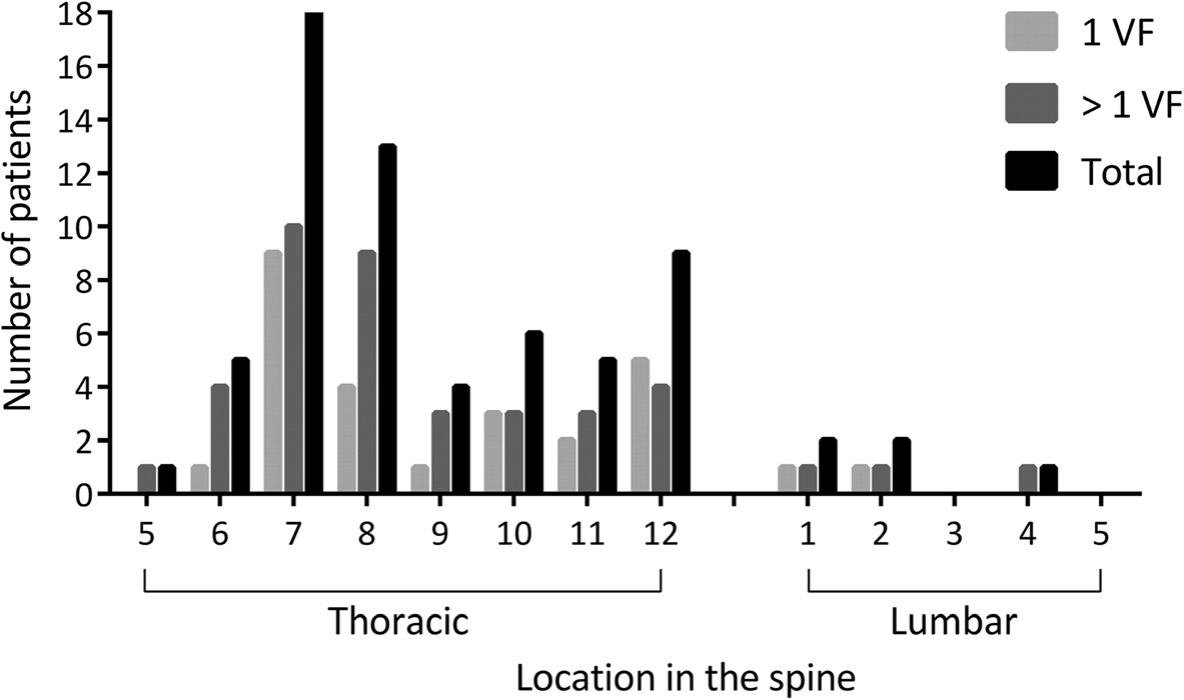
Prevalence of vertebral fractures (VFs) in the thoracic and lumbar spine of patients with spondyloarthropathy (SpA). The occurrence of VFs is represented as the absolute number of patients with SpA with VFs in either the thoracic (T5-T12) or lumbar part of the spine (L1-L5) (*black*). The absolute number of patients with SpA with only one VF located in the thoracic or lumbar spine is shown with *light gray bars. Dark gray bars* represent patients with SpA with more than one VF located in the thoracic or lumbar spine

By Rx, moderate to severe VFs were identified in 46 patients, of whom 25 (54.3 %) were men. Twenty patients (43.5 %) had more than one VF (18 had 2 VFs and 2 had 3 VFs), resulting in a total of 68 VFs. Of the 68 VFs, 62 (91.2 %) were located in the thoracic spine (Fig. 2). The prevalence was highest in IBD (22.2 %), followed by 12.2 % in AS and 10.0 % in PsA, and lowest in undifferentiated SpA (6.5 %).

Comparisons between patients with and without VF are shown in Table 2. Compared with patients without VF, patients with a VF were older (52.2 vs. 47.3 years, range 25–84 years; *p* < 0.01). They had a lower T-score in the femoral neck (−1.00 vs. −0.70; *p* < 0.05) and total hip (−0.74 vs. −0.27; *p* < 0.05), more peripheral joint disease (4.4 vs. 3.5; *p* < 0.05), a marginally higher classical (cervical + lumbar) mSASSS (11.7 vs. 7.0; *p* = 0.06), and a significantly higher thoracic mSASSS (16.2 vs. 9.8; *p* < 0.05).

**Table 2:** Basic characteristics of patients with spondyloarthritis without (*n* = 344) and with (*n* = 46) one or more radiographic vertebral fractures >25 %

Characteristics	Patients without VF	Patients with VF	*p* value^a^
	(n = 344)	(n = 46)	
Age, yr^b^	47.3 ± 11.6	52.2 ± 13.1	<0.01
Symptom duration, yr^c^	14.1 ± 10.4	18.5 ± 12.4	<0.01
Disease duration, yr^d^	10.5 ± 9.6	12.5 ± 12.1	ns
ESR, mm/h	9.2 ± 10.7	8.0 ± 8.1	ns
CRP, mg/L	4.0 ± 6.5	2.9 ± 3.8	ns
BASDAI	4.3 ± 2.3	4.7 ± 2.2	ns
BASFI	5.0 ± 8.5	4.9 ± 2.9	ns
ASDAS-ESR	2.2 ± 1.0	2.4 ± 0.9	ns
ASDAS-CRP	2.2 ± 1.0	2.3 ± 1.0	ns
OWD	1.3 ± 4.2	2.5 ± 5.5	0.09
mSASSS			
Cervical spine	3.4 ± 8.5	5.8 ± 13.2	0.10
Thoracic spine	9.8 ± 19.6	16.2 ± 25.1	<0.05
Lumbar spine	3.6 ± 7.6	6.0 ± 10.3	<0.07
Cervical + lumbar spine	7.0 ± 15.0	11.7 ± 21.6	0.06
Total spine	16.8 ± 32.8	27.9 ± 43.5	<0.05
T-score			
Lumbar spine	0.25 ± 1.70	0.13 ± 1.50	ns
Femoral neck	−0.70 ± 1.06	−1.00 ± 0.97	<0.05
Total hip	−0.34 ± 1.09	−0.59 ± 1.04	ns

*ESR* erythrocyte sedimentation rate, *CRP* C-reactive protein, *BASDAI* Bath Ankylosing Spondylitis Disease Activity Index, *BASFI* Bath Ankylosing Spondylitis Functional Index, *ASDAS* Ankylosing Spondylitis Disease Activity Score, *mSASSS* modified Stoke Ankylosing Spondylitis Spinal Score, *TNF* tumor necrosis factor, *ns* not statistically significant, *VF* vertebral fracture, *OWD* Occiput to wall distance

Characteristics of patients with spondyloarthritis (SpA) are represented as mean absolute number ± standard deviation

^a^Means of characteristics were compared between patients with SpA without and with VF using analysis of variance. *p* value <0.05 was considered statistically significant.

^b^Mean age ± standard deviation in years

^c^Mean symptom duration ± standard deviation in years; time since first symptoms

^d^Mean disease duration ± standard deviation in years; time since diagnosis

Compared with patients with an mSASSS score of 0 at the level of BMD measurement (L2-L4), patients with mSASSS scores >0 had a significantly higher T-score in the spine (0.47 vs. 0.12; *p* = 0.50). In contrast, the T-score was significantly lower in the femoral neck (−1.01 vs. −0.60; *p* < 0.001) and in the total hip (−0.65 vs. −0.22; *p* < 0.001).

**Fig. 3:**
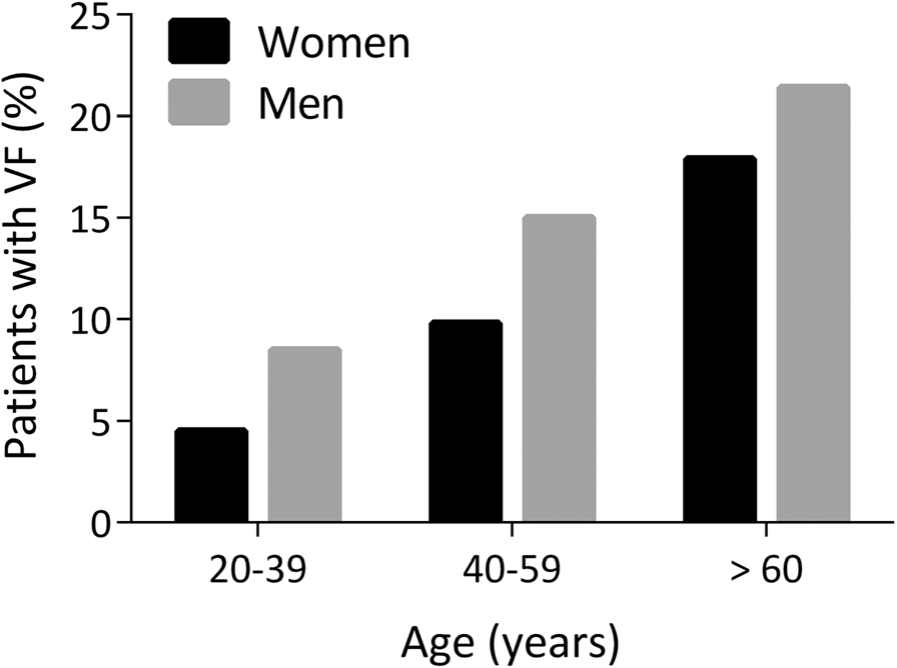
Prevalence of vertebral fractures (VFs) according to age and sex of patients with spondyloarthropathy (SpA). The prevalence of VFs is shown as the percentage of men (*gray bars*) or women (*black bars*) of the total number of patients with SpA with VFs in three different age groups. Age groups were 20–39 years, 40–59 years, and 60 years and older

Univariable logistic regression analysis revealed the OR for VF (based on the significant differences in Table 2): age (OR 1.35 per 10-year increase, 95 % CI 1.08–1.62) (Fig. 3), duration of symptoms (OR 1.35 per 10-year increase, 95 % CI 1.08–1.61), thoracic mSASSS (OR 1.13 per 10-point increase, 95 % CI 1.00–1.25), and femoral neck T-score (OR 1.38 per 1 SD decrease, 95 % CI 1.01–1.88). In multivariable regression analysis including these factors, only age remained significant, with an OR of 1.35 (95 % CI 1.08–1.62) per 10-year increase in age.

A history of falls was reported by 15.1 % of patients, 17.4 % with and 14.8 % without a VF (not statistically significant). A history of trauma with subsequent acute back pain was reported by 15.2 % of patients, 15.2 % with and 2.6 % without a VF (*p* = 0.001). A history of trauma with subsequent chronic back pain was reported by 1.3 % of patients, 8.7 % with and 0.3 % without a VF (*p* = 0.001).

One patient had a history of spontaneous dorsal arch fracture at T10 without previous trauma, which was diagnosed only after bone scintigraphy and magnetic resonance imaging and that healed spontaneously without complications other than temporarily increased back pain. Three patients (0.8 %) had a history of VF with neurologic complications: two after a cervical fracture and one after a lumbar fracture.

The reliability of Rx and VFA for diagnosing VF was found to be κ = 0.898 (standard error 0.036; *p* < 0.001) (Table 3). Rx VF could not be diagnosed on the basis of VFA in six patients, because the VFA images were of insufficient quality. In two patients, VFs were diagnosed with the use of VFA, and the VFs were also present on radiographs but with a deformity of <25 % on Rx.

**Table 3:** Analysis of congruence between conventional radiography and Vertebral Fracture Assessment for diagnosing vertebral fractures

		Rx		
		VF	No VF	Total
VFA	VF	40	2	42
	No VF	6	342	348
	Total	46	344	390

*VF* vertebral fracture, *VFA* Vertebral Fracture Assessment

Patients with spondyloarthritis with or without VF who were diagnosed by radiography (Rx) or by VFA using dual X-ray energy absorptiometry are represented as absolute numbers. Agreement between VFA and Rx was excellent (κ = 0.898, standard error 0.036; *p* < 0.001). The diagnostic value of VFA in identifying Rx VF indicated sensitivity of 87 % [95 % confidence interval (CI) 74–95 %], specificity of 99 % (95 % CI 98–100 %), positive predictive value of 95 % (95 % CI 84–99 %), and negative predictive value of 98 % (95 % CI 96–99 %)

The analysis of the diagnostic value of VFA to detect VFs identified on Rx indicated sensitivity of 87 % (95 % CI 74–95 %), with specificity of 99 % (95 % CI 98–100 %), PPV of 95 % (95 % CI 84–99 %), and NPV of 98 % (95 % CI 96–99 %).

## Discussion

In this large survey of VFs in 390 men and women with SpA, we found >10 % prevalence of moderate to severe VFs, already before the age of 30 years and within 5 years of symptoms.

The pathophysiology of VFs in SpA is complex [13, 24–26]. In AS, markers of bone resorption are increased in active disease and are related to low BMD and bone loss in the femoral neck and in the spine [27, 28]. Markers of bone formation are mostly normal in AS and not correlated with BMD [27]. Low BMD and bone loss have been documented in the spine [by DXA and quantitative computed tomography (QCT)] and in the hip (by DXA) [29, 30]. Later in the course of AS, BMD is decreased in the hip (as shown by DXA) and within the vertebrae (by QCT), but not in the lumbar spine (by DXA). This is due to intervertebral syndesmophyte formation, periosteal bone formation with squaring of the vertebrae, ankylosis of the facet joints, and calcification of the perivertebral ligaments (by QCT) [29, 30].

Using high-resolution peripheral QCT, it has been shown that patients with AS who have osteoporosis also have a decrease in bone volume density and in cortical thickness in the distal radius and distal tibia, which correlated with trabecular volumetric BMD in the lumbar spine using QCT [31]. These studies suggest that patients with AS have a generalized loss of trabecular bone density. The clinical consequence is that this low BMD and bone loss are measurable by DXA in the spine and hip early in the disease, but only in the hip later in the disease course [24, 25].

Interestingly, in this study, the presence of VF was related to mSASSS and marginally to BMD measured by DXA in the femoral neck. This indicates that, in addition to a low BMD, an altered biomechanical performance of the vertebrae by the stiffening of the spine contributes to the occurrence of VF [13, 24, 25]. These results further support the European League Against Rheumatism (EULAR) recommendations on imaging in SpA, which state that in patients with axial SpA without syndesmophytes in the lumbar spine visualized by conventional Rx, osteoporosis should be assessed by hip DXA and anteroposterior spine DXA. In patients with syndesmophytes in the lumbar spine visualized by conventional Rx, osteoporosis should be assessed by hip DXA supplemented by either spine DXA in lateral projection or possibly QCT of the spine [32].

VFs do not occur uniformly along the spine, but, as shown in postmenopausal and senile osteoporosis, they occur more often in the midthoracic and thoracolumbar regions than elsewhere [33, 34], which is likely attributable to biomechanical factors. Apart from loading (body height, weight, muscle forces, movements such as bending), other factors play a role in VF, such as spinal curvature and the heterogeneity of BMD between vertebrae [35]. Furthermore, a wedged thoracic VF increases the biomechanical stresses in other vertebrae [36]. In view of the relationship between VF and mSASSS in this and other studies [14], stiffening of the spine in SpA also could contribute to VF risk. Segmental or generalized syndesmophyte formation (bamboo spine) transforms the flexible spine in a long stiff bone (with intravertebral osteoporosis as shown with computed tomography) with decreased biomechanical competence. Stiffening of the spine in AS can also explain why VFs in AS occur at unusual locations (cervical spine) with unusual characteristics (e.g., transdiscal and horizontal transvertebral fractures and fractures of the dorsal arch structures of the vertebrae, which was reported by one of our patients) and with severe neurologic deficits, which occurred in three patients (0.8 %) [24].

Patients with a VF more often had a history of acute and chronic back pain after trauma than did patients without a VF. However, this occurred only in a minority of patients with a VF (one of six patients), suggesting that most VFs in SpA are not the result of trauma. The clinical consequence is that a history of trauma helps to identify only a limited number of patients with a VF. According to the ASAS/EULAR recommendations, imaging of the spine is advocated in AS in cases of a significant change in the course of the disease [15, 16]. However, on the basis of the high prevalence of subclinical VFs, this recommendation could need adaption, as VFs can be diagnosed only when imaging of the spine is performed and can be present without a history of trauma or typical signs and symptoms of an acute fracture.

The agreement between digitized Rx and VFA in diagnosing VFs, as well as the high NPV [17–19], is of clinical interest. VFA missed six (12.5 %) of the radiographic VFs. This indicates that in the majority of patients with SpA, VFA will enable diagnosis of VFs using a much lower level of radiation than Rx. Earlier studies showed less congruent results, but this could have been due to measuring vertebral heights using a less reliable magnification loop on plain radiographic films instead of electronic aids on digitized radiographs in this study [18, 19].

In view of its high NPV, VFA allows selection of patients who will or who will not need Rx for diagnosing VFs [17–19, 23]. A prevalent VF is a strong predictor of future VFs and non-VFs. No fracture prevention studies are available in SpA. It is therefore indicated that patients with SpA and early bone loss, osteoporosis, and/or a prevalent VF should be considered candidates for antiresorptive drugs to prevent fractures.

The strength of this study is the large sample collection and the representativeness of the patient group in a peripheral non-academic rheumatology center, including a broad range of ages and representing both sexes. Interestingly, we found the overall prevalence of SpAs to be as common in women as in men. The dominant male prevalence in AS is well accepted. However, the gender distribution in our survey is in line with the finding that, in recent years, the gender ratio approached 1:1 in an AS patient survey in Germany [37] and in a population survey in the south of Sweden for SpAs [38]. This finding has been included in the ASAS mission statement on epidemiology of AS [39]. The reported data regarding prevalence of PsA according to gender is conflicting [38]. The incidence of Crohn’s disease is similar between women and men, but the prevalence of IBD with SpA is unknown [40].

The finding that VFA using DXA has high sensitivity, specificity, PPV, and NPV compared with Rx is another strength with possible clinical consequences for screening. It indicates that VFA can be used as a prescreening tool for the presence of a VF in SpA, limiting the use of radiographs to patients with a VF on VFA, or when VFA is not of adequate quality to evaluate VFs.

A limitation of the study is the small number of patients with PsA and IBD. Furthermore, we chose only VFs with a deformity >25 % and not smaller deformities, as this would have increased sensitivity but decreased specificity for diagnosing a VF [23]. Therefore, the prevalence of VFs is even higher in this population when mild deformities are also included [14]. Last, the relationship between a history of trauma and the presence of VF should be interpreted with caution, as there is a possibility of recall bias of traumas.

## Conclusions

Moderate to severe VFs are found in more than 10 % of patients with SpA, already before the age of 40 years in 5 % of women and 9 % of men and within 5 years of disease symptoms. Most VFs are located in the thoracic region, are subclinical, and are related to low femoral neck BMD and stiffening of the thoracic spine. VFA is helpful in selecting patients in whom Rx should be performed to diagnose a VF.

## Abbreviations

ANOVA: Analysis of variance
AS: Ankylosing spondylitis
ASDAS: Ankylosing Spondylitis Disease Activity Score
BASDAI: Bath Ankylosing Spondylitis Disease Activity Index
BASFI: Bath Ankylosing Spondylitis Functional Index
BMD: Bone mineral density
CI: Confidence interval
CRP: C-reactive protein
DXA: Dual-energy X-ray absorptiometry
ESR: Erythrocyte sedimentation rate
EULAR: European League Against Rheumatism
HLA-B27: Human leukocyte antigen B27
IBD: Inflammatory bowel disease
mSASSS: Modified Stoke Ankylosing Spondylitis Spinal Score
NPV: Negative predictive value
OR: Odds ratio
OWD: Occiput to wall distance
PPV: Positive predictive value
PsA: Psoriatic arthritis
QCT: Quantitative computed tomography
Rx: Radiography
SD: Standard deviation
SpA: Spondyloarthropathy
SQ: Semiquantitative
TNF: Tumor necrosis factor
VF: Vertebral fracture
VFA: Vertebral Fracture Assessment
